# A cross-national study of the association between natural resource rents and homicide rates, 2000–12

**DOI:** 10.1177/1477370816661741

**Published:** 2016-08-16

**Authors:** Paul B. Stretesky, Michael A. Long, Michael J. Lynch

**Affiliations:** Northumbria University, UK; Northumbria University, UK; University of South Florida, USA

**Keywords:** Ecological disorganization, inequality and homicide, natural resource curse, social disorganization

## Abstract

Countries that rely on natural resource rents (that is, the revenue generated from the sale of natural resources) may suffer from a variety of social problems. This exploratory study reviews the natural resource extraction literature to derive a ‘natural resource rents–homicide’ hypothesis. Data for 173 countries for the years 2000 to 2012 are examined to determine if there is a correlation between natural resource rents and homicide rates. Multilevel growth models suggest that natural resource rents are positively correlated with homicide rates within countries (level 1) but not between them (level 2). Importantly, the correlation between natural resource rents and homicide is strongest when natural resource rents are lagged. We conclude by suggesting that increasing natural resource rents may be counterproductive over the long run and sow the seeds for a future increase in homicide.

## Introduction

Recent studies have found that excessive dependence on natural resource extraction revenue tends to be associated with elevated levels of crime in local settings (for example, Archbold et al., 2014; Carrington et al., 2011; Price et al., 2014; Ruddell, 2011; Ruddell et al., 2014). The major implication of these studies is that higher rates of crime are the result of detrimental social processes that are set in motion by a sudden increase in extraction development followed by a heavy reliance on revenue generated from natural resources. As yet, however, community-level findings that postulate a relationship between natural resource revenue and crime have not been replicated cross-nationally (except see Collier and Hoeffler, 2004). This observation raises the following question: is there a cross-national correlation between revenue that is generated by natural resource extraction and crime? To help answer this question we study one type of crime (that is, homicide rates) and natural resource rents. Homicide is one of the most valid and reliable cross-national crime indicators (see Neapolitan, 1997), and natural resource rents (NRR) are often used in development studies as an indicator of the amount of revenue generated by natural resources (less the cost of extracting those resources) that comes from their sale in the international market (Collier and Hoeffler, 2005). As a result, we test the hypotheses that NRR (as a percentage of gross domestic product) and homicide rates are positively correlated between and within countries.^1^

The present study begins by exploring the theoretical justification for the NRR–homicide hypotheses. Specifically, to derive our hypotheses we rely upon two sets of literature. First, we explore community-level studies that document the relationship between extraction-related economic activity and crime. Second, we examine cross-national level research by drawing upon the ‘the natural resource curse’ lens that stipulates countries with high NRR face more development-related problems than countries with low NRR. Within that natural resource curse literature we pay particular attention to the idea that high NRR countries may potentially harm the economy and encourage civil violence. We employ a multilevel growth model to test the correlation between NRR and homicide for 173 countries for the years 2000 to 2012. We then conclude by reflecting upon our findings in the context of the environmental justice literature and the potential for future research.

Exploring the potential effect of resource extraction on crime cross-nationally is important for two reasons. First, policy-makers across the globe regularly make decisions about the pace and extent of resource extraction to generate NRR. Information about the potential homicide–NRR association might be useful to policy-makers and affect the kinds of economic policies they select. Second, if a relationship between NRR and homicide exists, then criminal justice agencies faced with implementing enforcement practices may consider how to use NRR information to better advocate for and distribute resources to combat crime (Archbold, 2015).

## Background

Extractive activities consist of the removal of raw materials from nature (for example, coal, oil, gas, timber and minerals). These extraction efforts are often promoted by companies and governments, and are designed to stimulate revenue for economic development purposes. Local and national governments that are dependent on high NRR may suffer adverse social and economic consequences. For instance, Adger (2000: 361) suggests that too much reliance on NRR increases vulnerability to external shocks such as the volatility of raw material commodity prices. Jacquet (2014: 8321) describes four general types of problem that occur from an over-reliance on NRR: ‘(1) rapid industrialization and loss of governance, (2) uneven distribution of costs and benefits, (3) conflict, and (4) social-psychological stress and disruption.’ These potential adverse outcomes have led a variety of sociologists and development scholars to investigate the relationship between high NRR economies and wellbeing in the case of timber (Fisher, 2001; Wunder, 2001), oil (Obi, 2010), coal (Perdue and Pavela, 2012) and mineral mining (Pegg, 2006). Together these wellbeing studies suggest that a variety of social problems, including homicide, are likely to occur when governments and policy-makers pursue high NRR to boost economic growth.

### Community studies

Carrington, Hogg and McIntosh (2011: 353) propose that communities that rely on high levels of NRR experience greater levels of violence. That is, extractive economies disrupt social life in such a way that crime is more likely to occur. As a result, NRR can threaten community norms and values and therefore break informal ties and harm community services (Carrington et al., 2011; see also England and Albrecht, 1984; Freudenburg and Jones, 1991; Hunter et al., 2002; Wilkinson, 1991). However, it is not just a matter of elevated NRR promoting crimes such as homicide. It is also a matter of rapid social change that may occur in areas of high NRR. For instance, as Durkheim suggested in the 19th century with his concept of *anomie*, rapid social change disrupts community organization and attenuates social bonds, creating a sense of normlessness in the community that often leads to increases in crime (Durkheim, 2014 [1902]; see also Messner, 1989, for an empirical application to homicide rates).

The idea of social disorganization is often used to describe the condition faced by communities that undergo a rapid transition to an NRR-based economy (Ruddell et al., 2014). The increase in urbanization, industrialization and immigration that occurs along with rapid expansion of NRR may break social bonds and threaten conventional community beliefs and values. This disorganization can then lead to an increase in crime and delinquency (Kubrin and Weitzer, 2003; Shaw and McKay, 1942). Jacquet (2014: 8321) makes the connection between natural resource extraction and crime, drawing directly on concepts used in the social disorganization literature, suggesting that the loss of close-knit social ties can result in a loss of institutions (church groups, neighbourhood associations, etc.) that historically provide informal control over social problems including crime, drug abuse and mental illness, instead requiring formal organizations such as the police and court system to address these problems. Ruddell et al. (2014) propose that social disorganization may increase during a natural resource boom, and that is what leads to observed increases in crime. Anecdotal observations appear to confirm Ruddell et al.’s (2014 4) supposition, as reports by some law enforcement officials and residents of high extraction communities suggest that a ‘violent element’ is attracted to the employment opportunities brought on by rapid resource extraction that promotes social disorganization. As a result of these changes, communities that quickly become reliant on NRR are sometimes called ‘energy boom towns’.

Energy boom towns may also face rapid population growth, industrialization and fly-in, fly-out (FIFO) patterns of predominately male workers (Ruddell et al., 2014). This pattern of community development disrupts the normal patterns of life in the mostly rural areas where extraction takes place. The new residents and FIFO workers often cause problems for long-time residents, such as unemployment, increases in social-psychological stress and substance abuse (Jacquet, 2014). The transient nature of the workforce that migrates into quickly developing NRR economies may even create stress for local services because those employed in the industry often work shifts around the clock, disrupting families where increases in violent crimes such as domestic abuse have been reported (Carrington et al., 2011).

As noted, the rapid growth of extraction revenue may attract people seeking various types of work that ‘disrupt *normal* patterns of interaction’ (Ruddell et al., 2014: 3). Not surprisingly, changes in community values also affects the way that law enforcement responds to crime, reducing community service functions and increasing law enforcement functions. That is, police become more focused on catching and arresting criminals while reluctantly allowing minor disorder to flourish (Archbold, 2015).

Several empirical studies draw upon the social disorganization thesis to test the relationship between NRR and crime. As previously noted, these studies generally indicate that high-NRR economies also tend to have elevated levels of crime (for example, Archbold et al., 2014; Ruddell et al., 2014). For instance, recent research by Price et al. (2014) uncovers a relationship between the number of gas extraction wells (which presumably correlate to higher levels of NRR) and crime across local communities. The relationship between NRR and crime at the community level of analysis has also been observed within other developed countries such as Australia and Canada (Carrington et al., 2011; Ruddell, 2011).

A few studies propose that there is no correlation between NRR and crime (Wilkinson et al., 1982). Ruddell et al. (2014), for instance, found that although increases in NRR activity were followed by increases in index crime, high-NRR communities had similar index crime rates to low-NRR communities. Thus, Ruddell et al.’s results indicate that, in the case of homicide rates, it is the change in NRR and not the absolute level of NRR that matters. In addition, Hunter, Krannich and Smith (2002) point out that, although communities that pursue high levels of NRR as an economic strategy could eventually recover from elevated levels of NRR-related crime, perceptions of crime would be most intense for those socially disadvantaged residents living in high-NRR communities.

In sum, previous community-level research highlights how elevated and increasing NRR can foster disorganization. In turn, disorganization weakens community social bonds and creates individual and community-level stress that produces the right conditions for crime, including violence and homicide.

### Cross-national studies

Whereas problems associated with natural resource extraction are largely studied at the community level, the focus of this research is cross-national. As community scholars note, however, the same social processes that disrupt and disorganize communities may also be at work within and across countries. When studying extraction in communities, Freudenburg (1992: 307) proposed that ‘newly industrializing nations [may also suffer from natural resource addiction because] extractive activities are seen as having the potential to open up entire regions to economic development’.

Cross-national literature on the problems associated with NRR as a form of development can be broadly broken down into those studies that focus on economic growth and those that focus on civil unrest and violence. In 1990, Auty suggested that some countries experience the ‘curse of natural resources’.^2^ Like community-level research, the natural resource curse hypothesis proposes that development problems are likely to arise in economies that rely heavily on NRR.^3^ Shortly after Auty developed this thesis, Sachs and Warner used empirical data to study the effects of the resource curse, noting specifically that a ‘surprising [feature] of modern economic growth is that economies with abundant natural resources grow less rapidly than natural-resource-scarce economies’ (1995: 1). Their research demonstrates that developing countries with high levels of natural resource exports (for example oil, minerals and agriculture), expressed as a percentage of gross domestic product (GDP) in 1970, tended to experience slower economic growth over the next decade (see also Sachs and Warner 1997, 1999). Importantly, Sachs and Warner (1995, 1997) show that only 2 countries rich in natural resources (Malaysia and Mauritius) out of 19 did not experience a decline in GDP during the 1970s. The primary reasons that Sachs and Warner (2001) give for this general undesirable relationship is that countries rich in natural resources may be harming GDP by disrupting more traditional manufacturing growth. In other words, increases in natural resource extraction may quickly elevate wages and product prices within countries, making it harder for manufacturing to compete with other exports in the global marketplace. Sachs and Warner (2001: 833) note that, ‘[w]hatever the cause [for lower GDP], it is clear that we have not seen strong export-led growth in resource abundant economies’.

The implications of these economic problems have also led scholars to think about the relationship between NRR and homicide. Specifically, comparative researchers believe that NRR may produce a variety of social problems, including violence (Collier and Hoeffler, 2002). This more recent strand of research is similar to what is being proposed by community-level scholars – that the resource curse may create conflict and violence (Collier and Hoeffler, 2005; De Soysa and Neumayer, 2007; Ross, 2003; Skaperdas, 2002). Violence related to natural resources that occurs globally has garnered significant attention in the press, especially when related to conflicts over resources such as oil and minerals. In the popular press these conflicts are sometimes framed to highlight the violence behind their production and extraction (Eligon, 2011). For example, ‘blood diamonds’ or ‘conflict timber’ illustrate the social harm associated with the extraction of certain forms of natural resources (Mullins and Rothe 2008; Orogun, 2004; Van Solinge 2008). Lujala, Gleditsch and Gilmore (2005) also found ‘a diamonds curse’, indicating that, in the case of secondary diamonds, there was an increase in the likelihood of ethnic fighting.^4^ For example, murders and missing persons in Colombia are all too often associated with the capture of productive lands for the purpose of extracting palm oil (Borras and Franco, 2010a, 2010b). As a result, conflict over land associated with valuable natural resources may quickly lead to elevated murder rates (Myers, 1997). Moreover, as Downey, Bonds and Clark (2010) suggest, there are many different forms of extraction (for example mining and timber) that may encourage violence and murder in some countries.

There are some additional reasons that NRR may generate violence at a cross-national level. First, some researchers describe violence as a function of ‘looting rebels’ (Mildner et al., 2011). That is, in nation-states with weak governments, rebels can forcibly and violently take NRR as a method of funding political conflict. Collier and Hoeffler (2002: 625) conclude that NRR generate ‘low opportunity costs for rebellion and make civil war more likely’. Moreover, NRR reliance may challenge weak governments because law enforcement is strained and cannot provide protection in areas that can potentially generate high amounts of resource revenue. Thus, as Ross (2003: 25) contends, ‘this opens the door for criminal gangs, warlords, and rogue military officers’.

To date, there is only one academic study that examines the association between NRR and homicide rates at the cross-national level (Collier and Hoeffler, 2004). Specifically, Collier and Hoeffler (2004) studied deliberate killings and civil war deaths as a function of NRR.^5^ Collier and Hoeffler (2004) hypothesize that civil war deaths and homicides are likely to share predictors (that is, independent variables). The researchers conduct a cross-sectional analysis that illustrates important differences between the dependent variables. Specifically, they find that both ethnic dominance and NRR are positively correlated with the risk of civil war and that neither variable is correlated with homicide. Nevertheless, Collier and Hoeffler (2004) conclude that this finding requires further empirical investigation.

In sum, the literature on the impact of natural resource extraction and the resultant rents suggests the following two hypotheses, which we test in this article.

**Hypothesis 1:** Annual increases in a country’s natural resource rents are associated with annual increases in the country’s homicide rents (that is, the within-country hypothesis).
**Hypothesis 2:** Countries with higher average natural resource rents will have higher homicide rates compared with countries with lower natural resource rents (that is, the between-country hypothesis).

## Methods

We examine the cross-national correlation between NRR and homicide rates using multilevel models for change. This procedure models one dependent variable and multiple independent variables (Rabe-Hesketh and Skrondal, 2008; Raudenbush and Bryk, 2002). Multilevel change models are particularly appropriate for examining the relationship between NRR and homicide because they allow for the simultaneous estimations of year-to-year changes within individual countries (level 1) as well as between countries (level 2). Thus, we can assess the important question of whether NRR appear to facilitate homicide over time (within country), across space (between countries), or both. As per convention, we control for year in all of the statistical models.

We gathered data on natural resource extraction and other competing explanations of homicide for the years 2000 to 2012 for the 173 countries for which homicide and natural resource data were available over time (listed in Appendix 1). All of the variables used in our regression models of homicide are described below, with descriptive statistics for the overall sample presented in Appendix 2 and the between- and within-country variances listed in Appendix 3. We now turn to the description of the variables used in our models.

### Dependent variable

Our dependent variable is the homicide rate, measured as homicides per 100,000 persons. We focus our analysis on homicide rates because community and cross-national studies suggest that violence may be related to NRR and because homicide data are widely acknowledged to be the most valid and reliable cross-national indicator of violence (Neapolitan, 1997). As a result, it is possible to examine the variability in the homicide rate per country and per year (2000 to 2012).

**Figure 1.**
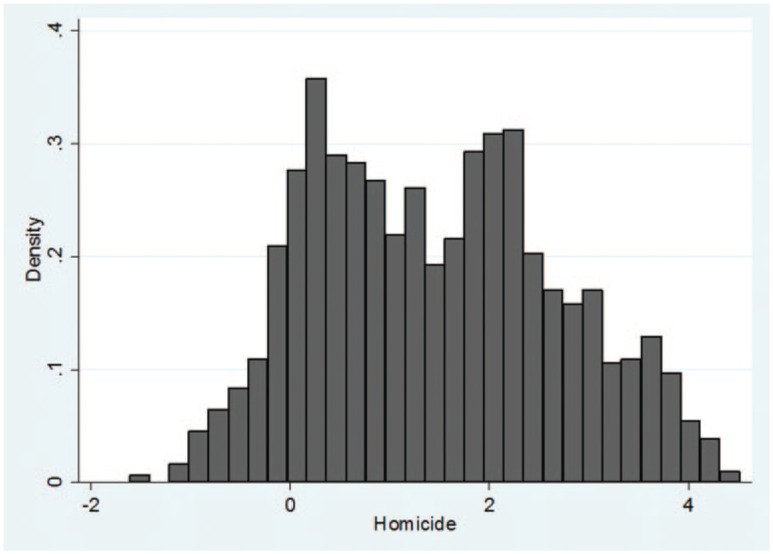
The natural log of homicide rates, 2000–12 (*n* = 828).

The total number of homicides per country, per year was extracted from the Global Study on Homicide 2013 (United Nations Office on Drugs and Crime, 2014), which compiles world homicide statistics over time. Data in this report are derived from criminal justice or public health systems and are verified through validity checks (see United Nations Office on Drugs and Crime, 2014: 110–11). These data intend to measure all recorded ‘unlawful deaths purposefully inflicted on a person by another person’ (United Nations Office on Drugs and Crime, 2014: 109). Because the distribution of homicide data is substantially non-normal across countries over time (skewness = 2.5, kurtosis = 7.7), we take the natural log of the variable to produce a better-fitting regression line and more normally distributed regression residuals. As noted in Figure 1, the distribution of the transformed homicide rate variable is relatively normal – even if slightly bimodal (skewness = 0.20, kurtosis = −0.79).

The natural log of homicide improves model fit substantially and reduced the standard error estimates at both level 1 and level 2, allowing for a better test of the natural resource curse–homicide hypothesis between and within countries.

### Natural resource extraction

We are interested in determining whether NRR is correlated with homicide rates between and/or within countries. Thus, we collected data on NRR for each country over time as a way to estimate the extent of extraction activity in a country as well as to measure how much a particular country relies on revenue generated from the sale of natural resources. NRR are one common measure of extraction relative to revenue. NRR data represent the surplus value or economic profits as a percentage of GDP that results from the sale of non-renewable natural resources (oil, gas, coal, minerals and timber). NRR, as a portion of GDP, are also often used as a measure of natural resource wealth in the natural resource curse literature (Collier and Hoeffler, 2005). As previously noted, as NRR increase, homicide should also increase because this form of economic development harms social life and leads to disruption and disorganization in communities and countries. Moreover, high NRR may promote political conflict between some countries at the national level. The NRR data are also highly skewed and peaked (skewness = 2.41, kurtosis = 4.36) because some countries rely heavily on NRR and others virtually not at all. As a result, we take the natural log of NRR because it helps to correct for the non-linear relationships that emerge between NRR and homicide (skewness = 0.71, kurtosis = 1.78). NRR data are available from the World Bank (http://data.worldbank.org; see also Hamilton and Clemens, 1998, for further discussion of NRR).

### Control variables

Several variables that might have an impact on the relationship between NRR and homicide must be controlled in the statistical analysis of NRR and homicides. Based on prior studies, GDP is one major variable that needs to be controlled in the analysis of homicide rates. GDP per capita across countries over time is collected from the World Bank and measured in thousands of US dollars per person. It is the market value of production, services and sales. The relationship between GDP and homicide is mixed in the literature, which finds positive, negative and no relationship between the variables (Pridemore and Trent, 2010). We also control for income inequality using the Gini index. The Gini index is obtained from the World Bank dataset and is frequently used as a measure of income inequality in social science research (http://data.worldbank.org). Gini is noted to be positively correlated with homicide rates in cross-national studies (Barber, 2006; Bjerregaard and Cochran, 2008; Nivette, 2011).

We control for three additional economic variables that were also collected from the World Bank. First, we control for the percentage of unemployed in each country each year since this variable has also been linked with homicide rates (Huang, 2001). That is, as unemployment increases across countries, so does homicide. Second, infant mortality is controlled when testing the relationship between NRR and homicide. Infant mortality measures the number of infants who die before the age of 1 per 1000 live births. Infant mortality is an indicator of poverty that should be positively associated with homicide rates between and within countries. Finally, foreign direct investment (FDI) is controlled and is an indicator of the direct investment of equity capital and the reinvestment of earnings into the economy as a proportion of GDP. Direct investment is typically not examined in homicide studies, but could drive NRR that impact on homicide rates. As a result, we examine the relationship between direct investments before NRR are included in the analysis to see if it is a potential mechanism that helps specify the relationship between NRR and homicide.

In addition to economic controls, we include five population indicators, one indicator of governance and one regional indicator. The percentage of the population in each country residing in an urban area is obtained from the World Bank and controlled in the analysis because increased urbanization has been linked to elevated homicide rates in some cross-national studies (Fischer, 1975). It is also an indicator of social disorganization because it has been argued that increases in urbanization are related to increases in crime. Population, measured in billions of residents, is also controlled in the analysis and allows us to assess if more populated countries or countries that grow in population also experience higher homicide rates. To account for age structure and gender we include the percentage of the population aged 0–14 years, the percentage of the population aged 15–64 years and the percentage of the population that is female.^6^ Democracy is examined as a control variable and is measured using the report *Freedom in the World*, produced by Freedom House (https://freedomhouse.org/report-types/freedom-world). The database documents each country’s political freedom in a ‘freedom index’ over time, using a rating that ranges from ‘1’ (most free) to ‘7’ (least free). We reverse-coded the democracy variable so higher values represent countries that have higher political freedom. We also include a measure of corruption, the Corruption Perception Index (CPI; Transparency International, 2016). The CPI is measured on a scale ranging from 0 (highly corrupt) to 10 (very clean). We reverse-coded CPI so higher values represent higher levels of corruption. Finally, the region of the world in which a country is located is included only in the between effects portion of the analysis, divided into the seven categories available in the World Bank data (Sub-Saharan Africa, East Asia Pacific, Europe Central Asia, Latin America Caribbean, Middle East North and North Africa, North America and South America). This variable is entered into the analysis as a dummy variable and the reference category is Sub-Saharan Africa (see Appendix 2).

### Analysis

To test the hypothesis that there is a positive correlation between NRR and homicide rates we use data for the 173 countries listed in Appendix 1 for the years 2000–12. Table 1 presents multilevel growth model estimates that test the viability of the notion that NRR and homicide are related. As noted, level-1 variables allow us to estimate the values of each variable at a specific year, and the level-2 variables average the values of the variable (that is, a grand mean centred) for each country from 2000 to 2012. In Table 1 we estimate three multilevel growth models for homicide rates. The first model (Model 1) examines the relationship between a five-year lag of NRR and homicide controlling only for region and year. We estimate the five-year lag of rents for theoretical and empirical reasons. That is, we rely on the natural resource curse literature, which notes that the impact of any resource curse is delayed rather than immediate (Ross, 2003). Also important, as noted below, is that the five-year lag produces the best-fitting model of NRR and homicide. We return to the issue of lagging NRR below, however, suggesting that, if social disruption and disorganization intensify over time, the lag in NRR produces the strongest correlation and is theoretically justified. Model 2 displays results for the impact of the control variables on homicide. Model 3 combines Model 1 and Model 2 variables to show the impact of NRR on homicide while controlling for typical variables used in cross-national analysis.

**Table 1.** Multilevel growth model coefficients of cross-national homicide rate for all countries, 2000–12.

	Model 1	Model 2	Model 3
*Level 1: Within country*			
Natural resource rents (lag)	0.076 (0.019)***		0.040 (0.019)*
Infant mortality (per 1000 live births)		0.245 (0.268)	−0.563 (0.397)
GDP per capita (US$ 000)		−0.025 (0.020)	0.042 (0.034)
Foreign direct investment (as percent of GDP)		−0.344 (1.279)	0.711 (1.364)
Percent unemployed		−0.001 (0.004)	−0.002 (0.005)
Democracy (Freedom Index)		0.032 (0.017)	0.025 (0.021)
Population (billion)		−0.716 (0.941)	−0.028 (1.758)
Percent urban		0.031 (0.006)***	0.021 (0.011)
Corruption (Corruption Perception Index)		0.049 (0.019)**	0.049 (0.025)*
Percent aged 0–14 years		0.006 (0.024)	−0.049 (0.036)
Percent aged 15–64 years		0.047 (0.021)*	−0.001(0.033)
Percent female		0.039 (0.048)	−0.112 (0.060)
Year	−0.015 (0.004)***	−0.033 (0.006)***	−0.046 (0.009)***
*Level 2: Between countries*			
Natural resource rents (lag)	0.010 (0.036)		−0.011 (0.054)
Infant mortality (per 1000 live births)		−1.324 (0.523)*	−0.648 (0.598)
GDP per capita (US$ 000)		0.047 (0.092)	−0.004 (0.099)
Foreign direct investment (as percent of GDP)		12.762 (12.57)	11.60 (12.71)
Inequality index (Gini)		0.029 (0.010)**	0.031 (0.011)**
Percent unemployed		0.030 (0.011)**	0.031 (0.012)**
Democracy (Freedom Index)		−0.060 (0.045)	−0.030 (0.049)
Population (billion)		1.095 (1.010)	0.288 (1.832)
Percent urban		−0.029 (0.008)***	−0.019 (0.013)
Corruption (Corruption Perception Index)		0.011 (0.068)	0.033 (0.071)
Percent aged 0–14 years		0.119 (0.037)**	0.170 (0.047)***
Percent aged 15–64 years		0.040 (0.041)	0.079 (0.050)
Percent female		0.450 (0.087)***	0.579 (0.096)***
*Region (vs. Sub-Saharan Africa)*			
East Asia Pacific	−1.196 (0.211)***	−0.361 (0.360)	−0.372 (0.364)
Europe Central Asia	−1.361 (0.175)***	−0.330 (0.373)	−0.290 (0.379)
Latin America Caribbean	0.651 (0.192)**	0.481 (0.291)	0.552 (0.293)
Middle East North Africa	−1.751 (0.216)***	−0.832 (0.368)	−0.713 (0.387)
North America	−1.126 (0.553)*	0.313 (0.512)	0.377 (0.517)
South America	−0.954 (0.314)**	0.237 (0.348)	0.287 (0.353)
Constant	31.90 (7.925)***	32.32 (14.01)*	60.21 (18.83)**
−2LL	−246.354	−54.168	−29.14
AIC	516.708	174.336	128.279
BIC	573.264	332.594	281.938
*N*	823	894	596
No. of countries	169	112	111

*Notes*: *p < .05; **p < .01; ***p < .001 (two-tailed); standard errors are in parentheses.

### Missing data

As noted earlier, cross-national homicide data are the most available and reliable cross-national crime indicator. However, it still suffers from missing data, particularly when employing longitudinal analysis (Neapolitan, 1997). In order to add validity to our findings we examined the distribution of missing data in the dataset for patterns and then employed several modelling strategies.

The frequency of missing cases for each variable is reported in Appendix 4. It is clear that homicide rates have a substantial number of missing values (*n* = 915). This is potentially problematic, but it is the reality of modelling cross-national homicide rates longitudinally. Several independent variables (that is Gini, percent unemployed, democracy and corruption) also have a number of missing values. The Gini index has a very large percentage of missing values (72.48 percent), suggesting that perhaps it should be eliminated from the models. We did not include it in the level-1 portion of the models for this reason; however, because the level-2 models are averaged between effects, we were able to keep it in level 2. Recall, level-2 coefficients are the average effects of the variable over the time period under study, 2000–12. Since every country in the model had a least one value of the Gini index during that time period, there are no missing values of Gini at level 2. We next employed the Stata 13.1 command – *misstable pattern* – to the dataset, which produces an analysis of all the different permutations of missing values. The three most common patterns of missing data in the dataset are, (1) values of all variables except missing Gini (*n* = 504), (2) values of all variables except missing Gini and homicide (*n* = 388) and (3) values of all variables except missing Gini, homicide and corruption (*n* = 117). All other permutations of missing data patterns had fewer than 100 cases. As noted, missing Gini values do not affect the results because it is not included in level-1 results, and missing homicide values are a common limitation to cross-national homicide studies (Neapolitan, 1997). However, the majority of existing cross-national studies are cross-sectional; in our longitudinal study the missing values have less of an impact on the results because we are modelling change. For example, if a country is missing a value for the corruption indicator for 2001, the multilevel change model will ignore the missing value and model the corruption change for that country between 2000 and 2002. Additionally, there are not missing values at level 2 for any of the variables because they are average values for 2000–12.

To further validate our results we modelled the data in three ways. First, we used listwise deletion and removed any country from the analysis that had a missing value on any variable. We next restricted the results to cases that had no missing values of NRR. Finally, we used the mi suite of commands in Stata to impute all missing values of all the independent variables in the analysis. The findings for the NRR variable were very similar in each method of analysis, resulting in the coefficients having the same level of statistical significance in each approach. We report the models from the first approach (listwise deletion) here.

## Results

Model 1 (Table 1) suggests that NRR are related to homicide, but only within countries (level 1) and not between them (level 2). That is, within countries the correlation between the natural log of NRR and the natural log of the homicide rate is .076 (*p* < .001; two-tailed). More specifically, a one unit annual change in the log of NRR is associated with a .076 unit annual change in the log of the homicide rate. As a result, Model 1 (level 1) suggests that, when examining the bivariate association between NRR and homicide rates, the two variables are positively correlated within (and not between) countries.

Model 2 – the control variable model – finds that several predictors are significantly related to NRR. Increases in the percentage of the population residing in urban areas are associated with increases in homicide rates (level 1). Governments that become more corrupt within countries experience associated increases in homicide rates (level 1). Increases in the percentage of the population aged 15–64 years are associated with increases in homicide rates (level 1). Income inequality is also positively correlated with homicide rates across countries (level 2) and implies that countries with high levels of income inequality also tend to have higher homicide rates. Percentage unemployed, percentage 0–14 years and percentage female are all positively correlated with homicide rates between countries (level 2). Interestingly, GDP per capita is not significantly related to homicide rates either within or between countries. This is not surprising because cross-national studies of homicide have found negative, positive and no significant relationship between GDP per capita and homicide rates (Pridemore and Trent, 2010), indicating that this relationship is empirically unclear.

Level-2 (Model 2) results for infant mortality and percentage urban are unusual. Infant mortality, an indicator of poverty, is negatively related to homicide rates in Model 2 (level 2), a strange finding given that poverty should be related to homicide rates across countries (Pridemore, 2008). Model 2 also suggests an odd finding for percentage urban. Specifically, countries with higher percentages of population residing in urban areas have lower homicide rates compared with countries with smaller urban populations. The remaining control variables (FDI, democracy and region) do not appear to be correlated with homicide rates.

Importantly, Model 3 (Table 1) provides support for the position that NRR continue to be correlated with homicide even after controlling for important factors that may influence homicide rates across countries. That is, according to Model 3 (level 1) a one unit annual change in the log of NRR is associated with a 0.040 unit annual change in the log of the homicide rate. The addition of NRR to Model 2 also helps explain the odd findings for infant mortality and percentage urban (both at level 2). Specifically, infant mortality and percentage urban become statistically insignificant in Model 3 (level 2) when NRR are included in the model, suggesting the relationship in Model 2 may have been a result of model misspecification. In the case of infant mortality, this finding perhaps implies that NRR may influence both poverty *and* homicide – a result that is consistent with the natural resource curse literature. That is, the changes in the type of development matter when it comes to homicide rates. Importantly, then, it appears that, within countries when relevant predictors of homicide are included in the model, only the type of economic development (natural resource rents) is related to homicide rates at level 1.

The results for NRR at level 2 (Model 3) are also important. The correlation between NRR and homicide remains non-existent at level 2 (Model 3), signifying that it is the change in NRR as opposed to the level of NRR that matters. It is interesting to note that these results are similar to the Ruddell et al.’s (2014) community-level findings that there are no differences between local communities that engage in natural resource extraction and those that do not, but those communities that do engage in extraction observe increases in crime over time. Thus, local findings appear to be supported at the cross-national level in Table 1.

**Figure 2.**
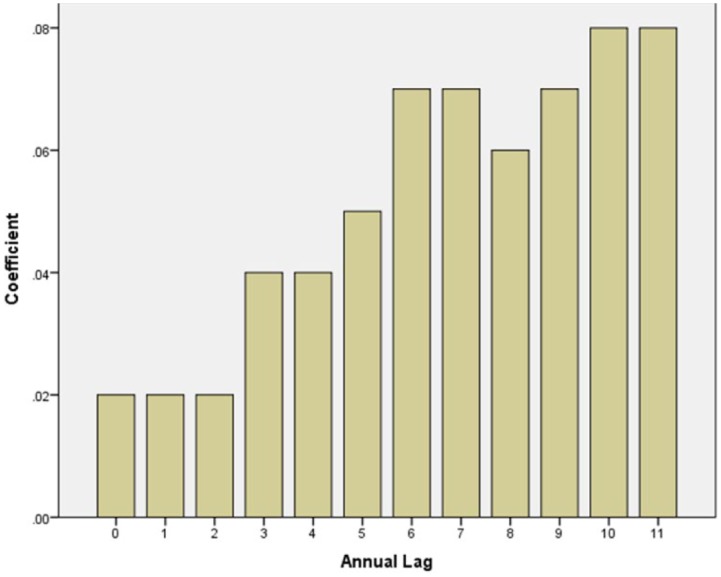
Relationship between natural resource rents (lagged by 0 to 11 years) and homicide rates.

The relationship between NRR and homicide requires some additional attention. That is, if NRR and homicide are correlated, how does that correlation emerge over time? As previously noted, we lagged NRR to examine the relationship between NRR and homicide. Figure 2 examines that lag in more detail – seeking to identify potential patterns in the relationship between NRR and homicide. An identified pattern in this relationship is important because it indicates whether countries may simply live with an increasing homicide rate over the short term. That is, if the relationship between NRR and homicide becomes weak over time, that might suggest that countries that adopt natural resource related development as a form of economic development simply need to ‘wait it out’. Since higher-extraction countries do not appear to have higher homicide rates, as indicated in Table 1, the within-country potential impact of increases in homicide rates may be short lived. Next, we investigate whether NRR is likely to have a delayed impact, by examining the correlation between its lag and homicide rates. The lagged analysis helps to determine if the potential impact of NRR on homicide is short lived (assuming such a relationship does indeed exist, as Table 1 suggests).

Figure 2 charts the level-1 coefficients for each lag of NRR on homicide in a multilevel model that controls for region, income inequality and the Gini index. The figure shows that the relationship between homicide rates and NRR is complex in that it emerges gradually over time. For example, at level 1 the relationship between that log of NRR and homicide – controlling for GDP, Gini and region – is not statistically significant until NRR is lagged by three years (coefficient = .04; *p* < .05). However, the association gradually increases so that by year 10 the association between NRR and homicide is .08 (*p* < .05). Thus, although it appears that having high levels of NRR is not a problem, countries that increase their reliance on NRR appear to create a significant problem over the long run with respect to increasing homicide rates. That is, it appears that the impact of increases in NRR matter for homicide rates in the future.

## Conclusion

Community- and country-level studies suggest that an overreliance on NRR may produce crime and violence. As an extension of this position we argued that a reliance on NRR may disrupt and disorganize social life and lead to higher levels of homicide. We tested the NRR–homicide hypothesis at the country level for the years 2000 to 2012 by examining the correlation between NRR and homicide rates. Our multilevel findings suggest that there is a correlation between NRR and homicide rates, but that correlation exists only with respect to changing reliance on NRR within countries. That is, the relationship between NRR and homicide does not appear between countries and high-extraction countries do not, on average, tend to have higher homicide rates. As a result, the NRR–homicide hypothesis suggests that within-country trends in NRR and homicide are positively correlated over time, whereas the between-country NRR–homicide hypothesis was rejected. This is an interesting finding because it suggests that rapid increases in NRR increase homicide, but that overall levels of NRR do not. To illustrate these different patterns it is helpful to briefly examine two specific countries. Guatemala’s homicide rate increased from 25.9 to 39.9 between 2000 and 2012, and its NRR increased from 2.4 percent to 4.2 percent over the same time frame. Guatemala’s NRR and homicide rates both almost doubled, demonstrating the within-group cross-national finding at a national level; that is, rapid increases in NRR are associated with rapid increases in homicide rates. In contrast, in 2012, Saudi Arabia’s NRR was 48.18 percent, indicating that almost half of the entire country’s GDP comes from NRR. However, in the same year, Saudi Arabia’s homicide rate was 0.8, one of the lowest of all countries in the dataset. As we found by failing to reject the between-country hypotheses, just because Saudi Arabia has a very high NRR value this does not mean that it will also have a high homicide rate. The effect of NRR comes when there is a rapid change in rents, not the overall value of the NRR.

The potential role of NRR in organizing homicide rates has implications for policy-makers who might be contemplating a shift to heavy reliance on NRR for development. First, although it is often assumed that natural resource extraction is unsustainable and leads to economic harm and environmental destruction over the long run, we believe there is an additional reason for caution. Specifically, although NRR may stimulate revenue in the short run it can also lead to increases in social problems, such as homicide, over the long run. There is no evidence to suggest that countries with high extraction rates have high homicide rates, but there is reason to suspect that social change – especially related to development – may contribute to changes in homicide. In some respects our findings regarding NRR sum up the essential nature of developmental change (that is, Durkheim, 2014 [1902]). That is, significant levels of social and economic change – and not the end result of that change – are what produce social problems such as homicide.

Second, if there is a relationship between NRR and homicide, then social institutions (for example, law enforcement) are likely to require more state support in countries that begin to shift their economies toward NRR-based economies (Archbold et al., 2014). However, extensively extractive economies are often viewed as overtly oppressive and many have been accused of using direct and forcible control to facilitate extraction. This situation is likely to raise human rights issues because state power may be directed toward facilitating state violence to increase NRR rather than reducing social violence to promote development. Although we do not have the space in this article, future research should examine whether development centred on NRR also promotes political violence, rather than the relatively non-political crime of homicide as it is measure in this study. Nevertheless, it could certainly be argued that the developed countries using extracted resources should be paying the costs associated with combating the additional homicide they create. This is even more important because countries that turn to NRR as a form of development are probably the least likely to adequately support state services that might serve to reduce violence. This particular situation could be viewed through an environmental justice lens, suggesting that environmental extraction produces harmful outcomes that are most likely to be imposed on less developed countries. In the end we argue that the role of natural resource extraction and the NRR–homicide hypothesis needs more serious consideration in cross-national studies of homicide because it may be counterproductive to the development process. We hope our findings have generated more interest in such research and recommend two areas for potential future study.

First, NRR are likely to vary by a country’s location in the international economy (Wallerstein, 1974), and underdeveloped as well as some developing nations are more likely to have economies linked to NRR than are developed nations. Scholars have argued that the majority of revenue generated from NRR does not help local economies; rather, because the extraction was funded by FDI, most of the profit leaves the local area and often the entire country (see Stiglitz, 2002: 67–73). This suggests that future research needs to examine the effects of foreign capital on social disorganization and homicide.

We not only find that countries with higher NRR have slightly higher homicide rates in general, but also suggest that within highly extractive countries an association between NRR and homicide is most likely to exist over time. As a result, it is possible that the effect of NRR on crime may harm some nations and not others, and that further analysis of this issue is required. Unfortunately, data on homicide are least likely to be collected in countries that are underdeveloped and have high NRR. We are not the first researchers to face data problems with respect to missing data among poorer countries with respect to cross-national homicide rates. Thus, future studies might look to various indicators of violence that better capture the impact of developmental change on homicide and violence more generally in less economically developed countries.

Second, our study examines the impact of NRR on homicide over only a relatively short period of time (13 years). That is, the true social impacts and cumulative nature of resource-based economies may not be realized for decades after the increases in NRR occur. Current limitations in cross-national homicide data (and control variables) do not allow for an expanded look at long-term cross-national homicide trends yet. However, as data continue to become available this relationship should be monitored. Although there are considerable calls for alternative energy development and use across the globe, we anticipate that rates of extraction of coal, oil and gas will continue to increase for some time because the demand for energy in the global economy has shown few signs of contracting in the near future.

## Appendix 1

**Table 2.** List of countries in the analysis (*N* = 173).

Afghanistan	Equatorial Guinea	Mexico	Tajikistan
Albania	Eritrea	Micronesia, Fed. Sts.	Tanzania
Algeria	Estonia	Moldova	Thailand
Angola	Ethiopia	Mongolia	Timor-Leste
Argentina	Fiji	Montenegro	Togo
Armenia	Finland	Morocco	Tonga
Aruba	France	Mozambique	Trinidad and Tobago
Australia	Gabon	Namibia	Tunisia
Austria	Gambia, The	Nepal	Turkey
Azerbaijan	Georgia	Netherlands	Turkmenistan
Bahamas, The	Germany	New Zealand	Uganda
Bahrain	Ghana	Nicaragua	Ukraine
Bangladesh	Greece	Niger	United Arab Emirates
Barbados	Guatemala	Nigeria	United Kingdom
Belarus	Guyana	Norway	United States
Belgium	Haiti	Oman	Uruguay
Belize	Honduras	Pakistan	Uzbekistan
Benin	Hong Kong SAR, China	Panama	Vanuatu
Bhutan	Hungary	Papua New Guinea	Venezuela, RB
Bolivia	India	Paraguay	Vietnam
Bosnia and Herzegovina	Indonesia	Peru	Yemen, Rep.
Botswana	Iran, Islamic Rep.	Philippines	Zambia
Brazil	Iraq	Poland	Zimbabwe
Brunei Darussalam	Ireland	Portugal	
Bulgaria	Israel	Qatar	
Burkina Faso	Italy	Romania	
Burundi	Jamaica	Russian Federation	
Cabo Verde	Japan	Rwanda	
Cambodia	Jordan	Samoa	
Cameroon	Kazakhstan	Sao Tome and Principe	
Canada	Kenya	Saudi Arabia	
Central African Republic	Kiribati	Senegal	
Chad	Kosovo	Serbia	
Chile	Kuwait	Seychelles	
China	Kyrgyz Republic	Sierra Leone	
Colombia	Lao PDR	Slovak Republic	
Comoros	Latvia	Slovenia	
Congo, Dem. Rep.	Lebanon	Solomon Islands	
Congo, Rep.	Lesotho	South Africa	
Costa Rica	Liberia	South Sudan	
Croatia	Libya	Spain	
Cyprus	Lithuania	Sri Lanka	
Czech Republic	Luxembourg	St. Lucia	
Denmark	Macao SAR, China	St. Vincent and the Grenadines	
Djibouti	Macedonia, FYR	Sudan	
Dominica	Madagascar	Suriname	
Dominican Republic	Malawi	Swaziland	
Ecuador	Malaysia	Sweden	
Egypt, Arab Rep.	Mali	Switzerland	
El Salvador	Mauritania	Syrian Arab Republic	

## Appendix 2

**Table 3.** Descriptive statistics for variables in the analysis.

Variable	Mean	Standard deviation
Natural log of homicide	1.42	1.24
Natural log of natural resource rents	1.33	1.89
GDP per capita (US$ 000)	1.00	1.58
Gini	40.46	9.99
Infant mortality (per 1000 live births)	33.20	29.25
Percent unemployed	9.04	6.34
Foreign direct investment (US$ 000)	0.01	0.01
Percent urban	53.91	23.23
Population (billion)	0.04	0.14
Democracy (Freedom Index)	4.64	2.09
Corruption (Corruption Perception Index)	6.86	2.14
Percent aged 0–14 years	30.56	10.83
Percent aged 15–64 years	62.10	7.06
Percent female	50.02	2.86
Region	Percent countries in region	
East Asia & Pacific	13.87	
Europe & Central Asia	27.75	
Latin America & Caribbean	17.34	
Middle East & North Africa	10.98	
North America	1.16	
South Asia	4.05	
Sub-Saharan Africa	24.86	

## Appendix 3

**Table 4.** Between and within variance.

Variable	Between	Within
Natural log homicide	1.26	0.07
Natural log resource rents	5.02	0.18
GDP per capita (US$ 000)	2.21	0.26
Gini	87.80	6.86
Infant mortality (per 1000 live births)	820.25	40.20
Percent unemployed	36.60	3.76
Foreign direct investment (US$ 000)	0.00004	0.0001
Percent urban	539.17	3.17
Population (billion)	0.02	0.0000004
Democracy (Freedom Index)	4.12	0.28
Corruption (Corruption Perception Index)	4.24	0.15
Percent aged 0–14 years	114.38	2.85
Percent aged 15–64 years	47.57	2.33
Percent female	7.92	0.27

## Appendix 4

**Table 5.** Description of missing values.

Variable	Number of missing cases	Percent missing
Natural log homicide	915	40.68
Natural log resource rents	177	7.87
GDP per capita (US$ 000)	21	0.93
Gini	1630	72.48
Infant mortality (per 1000 live births)	52	2.31
Percent unemployed	233	10.36
Foreign direct investment (US$ 000)	60	2.67
Percent urban	15	0.67
Population (billion)	0	0.00
Democracy (Freedom Index)	361	16.05
Corruption (Corruption Perception Index)	484	21.52
Percent aged 0–14 years	0	0.00
Percent aged 15–64 years	0	0.00
Percent female	0	0.00

*Note*: The dataset has a total of 2249 country-years, therefore the percentages in the table are derived from dividing the number of missing cases by 2249.

## Notes

1. We use the phrase ‘natural resource rents’ (NRR) throughout the article to refer to the amount of revenue generated from the extraction and sale of natural resources. Other terms sometimes used for this in the literature include ‘natural resource wealth’, ‘natural resource income’ and ‘natural resource revenue’. We use the phrase ‘natural resource extraction’ only when referring explicitly to the act of extraction.

2. That term was later modified and is now known as ‘the natural resource curse’ (see Frankel, 2010).

3. Gelb (1988) also examined this idea through the study of oil-producing states. Specifically, he examined how oil revenues in the 1970s helped and harmed developing oil exporters.

4. Although there is considerable support for the relationship between natural resource wealth and violence, not all researchers find that relationship. For instance, Brunnschweiler and Bulte (2009) discovered that natural resource riches do not increase violence for the 97 countries they studied between 1960 and 2004.

5. Collier and Hoeffler (2004: 5) note that, for their variables, ‘there is little variation over time [and as a result] there is a loss of efficiency in examining within country differences’. As a result, they relied on a cross-sectional analysis of homicide rates.

6. Many cross-sectional studies of homicide rates include the percentage of males aged 15–24 in their analyses to account for the fact that this is often seen as a group that commits a high level of crime. These data are not available for all of the countries in the analysis for all of the years. Therefore we include the age structure and the percent female variables as proxies.

## References

[bibr1-1477370816661741] Adger WN (2000) Social and ecological resilience: Are they related? Progress in Human Geography 24(3): 347–364.

[bibr2-1477370816661741] Archbold CA (2015) Established–outside relations, crime problems, and policing in oil boomtowns in Western North Dakota. Criminology, Criminal Justice Law, and Society 16(3): 19–40.

[bibr3-1477370816661741] Archbold CA Dahle T Jordan R (2014) Policing ‘The Patch’: Police response to rapid population growth in oil boomtowns in western North Dakota. Police Quarterly 17(4): 386–413.

[bibr4-1477370816661741] Auty RM (1990) Resource-based Industrialization: Sowing the Oil in Eight Developing Countries. New York: Oxford University Press.

[bibr5-1477370816661741] Barber N (2006) Why is violent crime so common in the Americas? Aggressive Behavior 32(5): 442–450.

[bibr6-1477370816661741] Bjerregaard B Cochran JK (2008) Want amid plenty: Developing and testing a cross-national measure of anomie. International Journal of Conflict and Violence 2(2): 182–193.

[bibr7-1477370816661741] Borras SM Franco J (2010a) From threat to opportunity? Problems with the idea of a ‘code of conduct’ for land-grabbing. Yale Human Rights and Development Law Journal 13(2): 507–523.

[bibr8-1477370816661741] Borras SM Franco J (2010b) Towards a broader view of the politics of global land grab: Rethinking land issues, reframing resistance. ICAS Working Paper Series No. 001. Initiatives in Critical Agrarian Studies, Land Deal Politics Initiative and Transnational Institute, The Netherlands.

[bibr9-1477370816661741] Brunnschweiler CN Bulte EH (2009) Natural resources and violent conflict: Resource abundance, dependence and the onset of civil wars. Oxford Economic Papers 61(4): 651–674.

[bibr10-1477370816661741] Carrington K Hogg R McIntosh A (2011) The resource boom’s underbelly: Criminological impacts of mining development. Australian and New Zealand Journal of Criminology 44(3): 335–354.

[bibr11-1477370816661741] Collier P Hoeffler A (2002) On the incidence of civil war in Africa. Journal of Conflict Resolution 46(1): 13–28.

[bibr12-1477370816661741] Collier P Hoeffler A (2004) Socio-economic determinants of homicide and civil war. CSAE WPS/2004-10). University of Oxford, Department of Economics.

[bibr13-1477370816661741] Collier P Hoeffler A (2005) Resource rents, governance, and conflict. The Journal of Conflict Resolution 49(4): 625–633.

[bibr14-1477370816661741] De Soysa I Neumayer E (2007) Resource wealth and the risk of civil war onset: Results from a new dataset of natural resource rents, 1970–1999. Conflict Management and Peace Science 24(3): 201–218.

[bibr15-1477370816661741] Downey L Bonds E Clark K (2010) Natural resource extraction, armed violence, and environmental degradation. Organization and Environment 23(4): 417–445. 21909231 10.1177/1086026610385903 PMC3169238

[bibr16-1477370816661741] Durkheim E (2014 [1902]) The Division of Labor in Society. Translated by Halls WD New York: Simon & Schuster.

[bibr17-1477370816661741] Eligon J (2011) Advocacy group quits coalition fighting sale of blood diamonds. New York Times, 6 12, A5.

[bibr18-1477370816661741] England JL Albrecht SL (1984) Boomtowns and social disruption. Rural Sociology 49(2): 230–246.

[bibr19-1477370816661741] Fischer CS (1975) Toward a subcultural theory of urbanism. American Journal of Sociology 80(6): 1319–1341.

[bibr20-1477370816661741] Fisher DR (2001) Resource dependency and rural poverty: Rural areas in the United States and Japan. Rural Sociology 66(2): 181–202.

[bibr21-1477370816661741] Frankel JA (2010) The natural resource curse: A survey. Faculty Research Working Paper Series. John F. Kennedy School of Government, Harvard University, Boston, MA.

[bibr22-1477370816661741] Freudenburg WR (1992) Addictive economies: Extractive industries and vulnerable localities in a changing world economy. Rural Sociology 57(3): 305–332.

[bibr23-1477370816661741] Freudenburg WR Jones RE (1991) Criminal behavior and rapid community growth: Examining the evidence. Rural Sociology 56(4): 619–645.

[bibr24-1477370816661741] Gelb A (1988) Oil Windfalls, Blessing or Curse. Oxford: Oxford University Press.

[bibr25-1477370816661741] Hamilton K Clemens M (1998) Genuine savings rates in developing countries. World Bank Economic Review 13(2): 333–356.

[bibr26-1477370816661741] Huang WSW (2001) A research note on reporting discrepancies in international homicide data. International Journal of Comparative and Applied Criminal Justice 25(2): 167–181.

[bibr27-1477370816661741] Hunter LM Krannich RS Smith MD (2002) Rural migration, rapid growth, and fear of crime. Rural Sociology 67(1): 71–89.

[bibr28-1477370816661741] Jacquet J (2014) Review of risks to communities from shale energy development. Environmental Science and Technology 48(15): 8321–8333. 24624971 10.1021/es404647x

[bibr29-1477370816661741] Kubrin CE Weitzer R (2003) New directions in social disorganization theory. Journal of Research in Crime and Delinquency 40(4): 374–402.

[bibr30-1477370816661741] Lujala P Gleditsch NP Gilmore E (2005) A diamond curse? Civil war and a lootable resource. Journal of Conflict Resolution 49(4): 538–562.

[bibr31-1477370816661741] Messner SF (1989) Economic discrimination and societal homicide rates: Further evidence on the cost of inequality. American Sociological Review 54: 597–611.

[bibr32-1477370816661741] Mildner S-A Lauster G Wodni W (2011) Scarcity and abundance revisited: A literature review on natural resources and conflict. International Journal of Conflict and Violence 5(1): 155–172.

[bibr33-1477370816661741] Mullins CW Rothe DL (2008) Gold, diamonds and blood: International state corporate crime in the Democratic Republic of the Congo. Contemporary Justice Review: Issues in Criminal, Social, and Restorative Justice 11(2): 81–99.

[bibr34-1477370816661741] Myers N (1997) Environmental refugees. Population and Environment: A Journal of Interdisciplinary Studies 19(2): 167–182.

[bibr35-1477370816661741] Neapolitan JL (1997) Cross-National Crime: A Research Review and Sourcebook. Westport, CT: Greenwood.

[bibr36-1477370816661741] Nivette AE (2011) Cross-national predictors of crime: A meta- analysis. Homicide Studies 15(2): 103–131.

[bibr37-1477370816661741] Obi CI (2010) Oil extraction, dispossession, resistance, and conflict in Nigeria’s oil-rich Niger Delta. Canadian Journal of Development Studies 30(1–2): 219–236.

[bibr38-1477370816661741] Orogun P (2004) Blood diamonds and Africa’s armed conflicts in the post-cold war era. World Affairs 166(3): 151–161.

[bibr39-1477370816661741] Pegg S (2006) Mining and poverty deduction: Transforming rhetoric into reality. Journal of Cleaner Production 14(3): 376–387.

[bibr40-1477370816661741] Perdue RT Pavela G (2012) Addictive economies and coal dependency: Methods of extraction and socioeconomic outcomes in West Virginia, 1997–2009. Organization and Environment 25(4): 368–384.

[bibr41-1477370816661741] Price M Basurto L Herzenberg S Polson D Ward S Wazeter E (2014) The shale tipping point: The relationship of drilling to crime, traffic fatalities, STDs, and rents in Pennsylvania, West Virginia, and Ohio. URL (accessed 16 July 2016): http://www.multistateshale.org/shale-tipping-point.

[bibr42-1477370816661741] Pridemore WA (2008) A methodological addition to the cross-national empirical literature on social structure and homicide: A first test of the poverty–homicide thesis. Criminology 46(1): 133–154.

[bibr43-1477370816661741] Pridemore WA Trent CLS (2010) Do the invariant findings of Land, McCall, and Cohen generalize to cross-national studies of social structure and homicide? Homicide Studies 14(3): 296–335.

[bibr44-1477370816661741] Rabe-Hesketh S Skrondal A (2008) Multilevel and Longitudinal Modeling Using Stata. College Station, TX: Stata Press.

[bibr45-1477370816661741] Raudenbush S Bryk AS (2002) Hierarchical Linear Models: Applications and Data Analysis Methods, 2nd edn. Thousand Oaks, CA: Sage.

[bibr46-1477370816661741] Ross M (2003) The natural resource curse: How wealth can make you poor. In: Bannon I Collier P (eds) Natural Resources and Violent Conflict: Options and Actions. Washington, DC: World Bank, 17–42.

[bibr47-1477370816661741] Ruddell R (2011) Boomtown policing: Responding to the dark side of resource development. Policing 5(4): 328–342.

[bibr48-1477370816661741] Ruddell R Jayasundara DS Mayzer R Heitkamp T (2014) Drilling down: An examination of the boom–crime relationship in resource based boom counties. Western Criminology Review 15(1): 3–17.

[bibr49-1477370816661741] Sachs JD Warner AM (1995) Natural resource abundance and economic growth. NBER Working Paper No. 5398, National Bureau of Economic Research Working Paper Series. Cambridge, MA URL (accessed 16 July 2016): http://www.nber.org/papers/w5398.

[bibr50-1477370816661741] Sachs JD Warner AM (1997) Natural resource abundance and economic growth, revised version. NBER Working Paper No. 5398, National Bureau of Economic Research Working Paper Series. Cambridge, MA URL (accessed 16 July 2016): http://academiccommons.columbia.edu/catalog/ac:138842.

[bibr51-1477370816661741] Sachs JD Warner AM (1999) The big push, natural resource booms and growth. Journal of Development Economics 59: 43–76.

[bibr52-1477370816661741] Sachs JD Warner AM (2001) Natural resources and economic development: The curse of natural resources. European Economic Review 45(4): 827–838.

[bibr53-1477370816661741] Shaw CR McKay HD (1942) Juvenile Delinquency and Urban Areas. Chicago, IL: University of Chicago Press.

[bibr54-1477370816661741] Skaperdas S (2002) Warlord competition. Journal of Peace Research 39(4): 435–446.

[bibr55-1477370816661741] Stiglitz J (2002) Globalization and Its Discontents. London: Penguin.

[bibr56-1477370816661741] Transparency International (2016) Corruption Perceptions Index. URL (accessed 16 July 2016): http://www.transparency.org/research/cpi/overview.

[bibr57-1477370816661741] United Nations Office on Drugs and Crime (2014) Global Study on Homicide 2013. United Nations Publication, Sales No. 14.IV.1. Vienna, Austria: Research Trend Analysis Branch, Division for Policy Analysis and Public Affairs, Office on Drugs and Crime.

[bibr58-1477370816661741] Van Solinge TB (2008) Eco-crime: The tropical timber trade. In: Siegel D Nelen H (eds) Organized Crime: Culture, Markets and Policies. New York: Springer, 97–112

[bibr59-1477370816661741] Wallerstein I (1974) The Modern World-System, Volume I: Capitalist Agriculture and the Origins of the European World-Economy in the Sixteenth Century. New York: Academic Press.

[bibr60-1477370816661741] Wilkinson KP (1991) The Community in Rural America. Westport, CT: Greenwood Publishing Group.

[bibr61-1477370816661741] Wilkinson KP Thompson JG Reynolds RR Ostresh LM (1982) Local social disruption and western energy development: A critical review. Sociological Perspectives 25(3): 275–296.

[bibr62-1477370816661741] Wunder S (2001) Poverty alleviation and tropical forests – What scope for synergies? World Development 29(11): 1817–1833.

